# Validation of Novel Image Processing Method for Objective Quantification of Intra-Articular Bleeding During Arthroscopic Procedures

**DOI:** 10.3390/jimaging11020040

**Published:** 2025-01-31

**Authors:** Olgar Birsel, Umut Zengin, Ilker Eren, Ali Ersen, Beren Semiz, Mehmet Demirhan

**Affiliations:** 1 Department of Orthopaedics and Traumatology, School of Medicine, Koc University, 34450 Istanbul, Turkey; ieren@ku.edu.tr (I.E.); mdemirhan@ku.edu.tr (M.D.); 2 Department of Electrical and Electronics Engineering, College of Engineering, Koc University, 34450 Istanbul, Turkey; uzengin19@ku.edu.tr (U.Z.); besemiz@ku.edu.tr (B.S.); 3 Department of Orthopaedics and Traumatology, Medical Faculty, Istanbul University, 34093 Istanbul, Turkey; ali.ersen@istanbul.edu.tr

**Keywords:** arthroscopy, bleeding, image processing, objective scoring, visual clarity

## Abstract

Visual clarity is crucial for shoulder arthroscopy, directly influencing surgical precision and outcomes. Despite advances in imaging technology, intraoperative bleeding remains a significant obstacle to optimal visibility, with subjective evaluation methods lacking consistency and standardization. This study proposes a novel image processing system to objectively quantify bleeding and assess surgical effectiveness. The system uses color recognition algorithms to calculate a bleeding score based on pixel ratios by incorporating multiple color spaces to enhance accuracy and minimize errors. Moreover, 200 three-second video clips from prior arthroscopic rotator cuff repairs were evaluated by three senior surgeons trained on the system’s color metrics and scoring process. Assessments were repeated two weeks later to test intraobserver reliability. The system’s scores were compared to the average score given by the surgeons. The average surgeon-assigned score was 5.10 (range: 1–9.66), while the system scored videos from 1 to 9.46, with an average of 5.08. The mean absolute error between system and surgeon scores was 0.56, with a standard deviation of 0.50, achieving agreement ranging from [0.96,0.98] with 96.7% confidence (ICC = 0.967). This system provides a standardized method to evaluate intraoperative bleeding, enabling the precise detection of blood variations and supporting advanced technologies like autonomous arthropumps to enhance arthroscopy and surgical outcomes.

## 1. Introduction

Achieving adequate visual clarity is critical during arthroscopic shoulder surgery. Several factors can hinder visualization during shoulder arthroscopy including bleeding, patient positioning, portal placement, fluid extravasation and abnormal anatomical structures. Despite advancements in modern imaging technology that provide ultra-high-resolution images and effective lighting, bleeding remains the primary challenge to maintaining smooth surgical workflows. Various techniques are employed to manage this issue, including the precise control of bleeding vessels, the use of hypotensive anesthesia, and pressure-regulated pump systems [1,2,3,4].

Maintaining sufficient pressure in the surgical field to occlude small vessels is essential for proper visualization during arthroscopic procedures. Morrison et al. determined that a pressure differential of 49 mmHg between systolic blood pressure (SBP) and subacromial space pressure (SASP) is required to ensure visual clarity [3]. To achieve this, higher pump pressures are used to distend the joint, improving visibility but often causing extravasation and soft tissue swelling, which complicates the surgery [5,6]. More recently, modifying the chemical composition of the irrigation solution has been explored to control bleeding, including the use of tranexamic acid to stabilize thrombosis and epinephrine for inducing intra-articular vasoconstriction [4,7,8,9].

Despite numerous interventions aimed at enhancing visualization during shoulder arthroscopy, no objective method exists to evaluate the effectiveness of these techniques. Even high-level randomized controlled studies assessing the impact of tranexamic acid on bleeding are limited by the reliance on visual assessments made by the human eye, which are inherently subjective [9,10,11,12,13,14,15]. Jensen et al. previously proposed quantifying intra-articular bleeding by measuring hemoglobin concentration in the irrigation fluid, a method that poses challenges in collecting all extravasated fluid [12]. Intra-articular bleeding has often been quantified using subjective scoring systems, with a 5-point visual analog scale being the most applied method in the literature. Nicholson et al. reported that intravenous tranexamic acid did not significantly improve the surgeon’s ability to maintain lower pump pressure during arthroscopic rotator cuff repair [13]. In contrast, Ersin et al. found that IV tranexamic acid improved visual clarity and reduced the need for high pump pressure [11]. The discrepancy between these studies can be attributed to two factors: first, surgeons lacked an objective method to quantify bleeding, and second, the 5-point scale is insufficiently precise to detect small but clinically relevant changes.

Amid ongoing controversies, the literature on improving arthroscopic visualization is filled with unresolved debates. Contrary to common belief, the relationship between intraoperative arterial blood pressure and visual clarity has never been objectively established. To demonstrate such a relationship, visual clarity must be scored every minute for a corresponding blood pressure value obtained from continuous blood pressure monitoring. Considering the average duration of an arthroscopic procedure and the need for numerous repetitions of the measurement, the need for a standardized image processing system is evident.

Moreover, factors such as patient positioning, brachial plexus block, optimal inflow rates, and the use of cannulas may all influence visualization [4,16,17,18]. However, these variables remain difficult to study due to the absence of standardization and a precise measurement tool. A multitude of experiments required for each variable, while the bleeding is scored by a surgeon, would be time-consuming and, more importantly, subjective, as it is influenced by human judgment and behavior. Based on these motivations, there is a compelling need to develop an objective, standardized, and reproducible method to replace inherently subjective surgeon-rated visual analog scores.

This validation study investigates whether a color recognition system can accurately identify bleeding and assist surgeons in objectively quantifying intra-articular blood volume. To that aim, we hypothesized that the bright red of bleeding could be distinguished from the pale grayish-white environment of the glenohumeral joint and subacromial spaces using an image processing analysis system. Recent advancements in image processing have paved the way for the numerical expression of the image sections taken from the videos in various color spaces. While analyzing videos, like those used in arthroscopic surgery, image frames can be converted into these color spaces to quantify and interpret colors more precisely. While the **RGB** (red, green, blue) space is the most common color space used in image processing, it cannot fully distinguish the subtle differences between similar shades of color, particularly within the same spectrum (such as different reds) [19]. On the other hand, **HSV** (hue, saturation, value) color space separates color (hue) from intensity (value/brightness), representing color more like how humans perceive it [19,20]. As it has the potential to distinguish shades of the same color, differentiating various intensities of red in a surgical video could potentially benefit from the HSV space. Finally, **CIELAB** (lightness, A: red/green coordinate, B: yellow/blue coordinate) color space is designed to approximate human vision and covers a wider range of colors relative to RGB. As it has the potential to capture subtle changes in color, it could potentially identify small amounts of blood in a surgical video, which may appear as varying shades of red or pink [20].

It has been observed that there are very few studies in the literature on this subject and that the existing studies are unlikely to achieve the expected sensitivity and accuracy in arthroscopic procedures. The identification of bleeding through visual technology has been previously utilized in laparoscopic surgery [21,22,23]; however, the visual characteristics of bleeding vary between laparoscopic and arthroscopic systems. In laparoscopy, blood has a more uniform texture in an inflated setting, whereas in arthroscopy, blood behaves like a red dye, gradually mixing with the arthroscopic fluid, complicating image processing [24]. On the other hand, Tuijthof et al. leveraged an RGB-based algorithm on the arthroscopy images; however, they solely focused on the acute bleeding episodes overlooking constant low-intensity leakage into the joint [25]. Finally, Barnes et al. focused on the general image quality metrics including brightness, local contrast, and image entropy along with the redness for representing the blood [26]. Using 11 s long frames, they developed a four-point scale from poor to excellent view; however, the study was limited by the narrow grading range, lack of thresholding and long window lengths missing intra-frame variations.

To address these limitations and fill the gap in the literature, we propose a novel analysis framework leveraging HSV and CIELAB color spaces to develop a frame-wise bleeding scoring system. The contributions of the study can be listed as follows:

- We propose a novel thresholding formula leveraging the combination of HSV and CIELAB parameters that can best mimic the human eye to develop a gradient-based (1 to 10) scoring system capable of detecting small changes at low levels in the articular space.
- By selecting three random frames from each second and comparing the average score of each second to the previous three, our system suppresses the false positives caused by transient red tissue artifacts.
- Rather than focusing solely on bleeding episodes, our system outputs both a frame-wise score and a total bleeding score for the entire surgical procedure, which provides instantaneous and overall bleeding assessment at the same time.

The remainder of the paper is organized as follows: Section 2 details our hypothesis, the formulation process, and the validation study. Section 3 presents the results. Section 4 discusses the findings from a clinical perspective, compares them with the existing literature, and addresses the study’s limitations and potential directions for future work. Finally, Section 5 summarizes the key findings and provides concluding remarks.

## 2. Materials and Methods

### 2.1. Hypothesis

Our hypothesis is based on the observation that tissues encountered in arthroscopic procedures predominantly appear in pale white, beige, and grayish tones, while bleeding distinctly stains the arthroscopic fluid a bright red, representing the opposite end of the color spectrum. Although intense bleeding is easy to identify due to its strong visual contrast, our goal was to develop a gradient-based scoring system capable of detecting very small amounts of blood in the articular space. To achieve this, a thresholding method was necessary to exclude potential confounders, such as inflammation, which may appear in various shades of red, and muscle tissue, which occasionally appears pale pink. As more blood dissolves in the arthroscopic fluid, the pixels in the image gradually shift toward the red spectrum. We hypothesized that a combination of a specific ’redness ratio’ and saturation levels could reliably estimate the intensity of bleeding into the fluid, regardless of the total blood volume in the joint.
**Algorithm 1:** Pseudocode for video clip scoring
**Input:**current 3-second-long video clip video(t) previous 3-second-long video clip video(t−1)

**Output:**bleeding score for the current 3-second-long video clip score(t)
**1.****for** each 1-second-long segment(i) in video(t) **do****2.**
choose three random frames from segment(i)
**3.**
**for** each selected frame in segment(i) **do****4.**

remove the black area around **5.**

extract H, S, V parameters through HSV-space conversion**6.**

extract L, A, B parameters through CIELAB-space conversion**7.**

insert values into the scoring formula**8.**

calculate the pixel ratio meeting the thresholding criteria**9.**

map this value to the range of [1, 10]**10.**
calculate *score*$\left(t\right)$ as the average score for three frames**11.**
**if**abs(score(t)–score(t−1))>3:**12.**

 choose another set of three random frames and **repeat** steps **3–10****13.**
**else:**
**14.**

**return** 
$score\left(t\right)$


### 2.2. Creation of the Formula

A novel frame-wise analysis pipeline was developed and tested in the Python programming platform (version 3.9) and the application for patenting was dated in May 2023. This image processing system was designed to count the number of pixels that met specific threshold criteria and compute their ratio to the total pixel count, assigning a score to each image. To increase the accuracy and reduce the error, we implemented HSV (hue, saturation and value) and CIELAB (lightness, A: red/green coordinate, B: yellow/blue coordinate) color spaces along with the mean saturation and mean A values simultaneously. At the end of an extensive trial and error procedure that involved more than 30 h of video footage and was supervised by experienced surgeons, thresholding limits as well as the ideal combination of parameters that would best mimic the human eye were formulized. 

**Figure 1:**
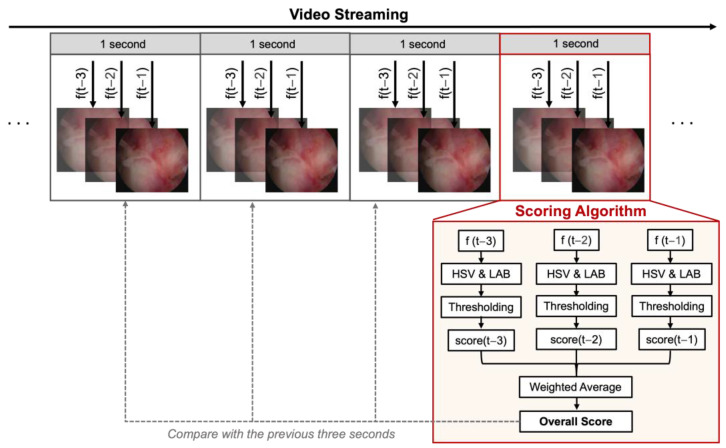
An image processing system was designed to count pixels meeting threshold criteria and calculate their ratio to the total, assigning a score to each image. It evaluates three random frames per second (from 24 fps) and compares each second’s average score to the previous three seconds’ scores to suppress the effect of spontaneous red tissue artifacts.

The pseudocode for the proposed algorithm and the block diagram are presented in Algorithm 1 and Figure 1, respectively. The system evaluates three random frames from each second (out of 24 frames per second), comparing the average score of each second to the previous three. Floating clot pieces or coagulated tissue that are closely visualized can instantaneously deceive the system imitating a local bleeding. However, the rule of comparing the score to three previous ones aims to limit the effect of a spontaneous red tissue artifact on the score. To achieve this, each video was duplicated to create a virtual “previous three seconds”, ensuring consistency and simulating real-time analysis. This approach allowed the system to effectively analyze a 6 s video consisting of two 3 s segments, replicating the evaluation of the original video’s final three seconds. Considering that even acute intense bleeding is gradual and should not increase the score abruptly, the system is programmed to check for another set of three frames if there is more than a 3-score increase within a second.

### 2.3. Validation Study

**Figure 2:**
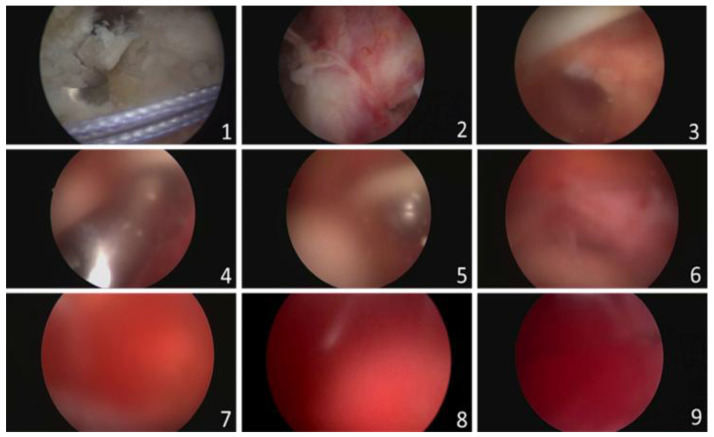
Grading scale from 1 to 9 according to the scores given by the fourth surgeon. This tutorial is created for training the raters, indicating the gradually increasing amount of bleeding into the surgical area. Note that the redness, saturation, and blur are increasing from 1 to 9.

Two hundred video clips, each with a duration of 3 s, were prepared by a fellowship trained shoulder surgeon, making sure to include 20 video clips for each score from 1 to 10, based on his subjective consideration. Sample images with different scores are provided in Figure 2 (and additionally as Supplementary Material Figure S1). The video clips were selected from video records of the 15 previously operated arthroscopic rotator cuff repair procedures (9 females and 6 males with an age = 64.6 ± 4.7), with essential care given to visual homogeneity. This means that the score was consistently the same for every second of the video clip. The video clips that composed our dataset were particularly selected among the recordings of rotator cuff repair patients because this procedure included the intervention of subacromial fossa, which contains the highly vascularised inflamed bursal tissue. Three fellowship trained senior shoulder surgeons with a minimum of 10 years of experience in shoulder arthroscopy were instructed on the color metrics and grading of the bleeding using samples of one video from each score before evaluating the videos. Two evaluations were conducted with a 2-week interval for assessing intraobserver reliability. All videos were captured with the same video record unit (Karl Storz, AIDA WD 250) using the same adjustments (HD, mode: CLARA + CHROMA) that were utilized anonymously by all three surgeons. The interobserver agreement and intraobserver consistency were noted. The system subsequently scored 200 video clips, and its results were compared to the average score of the three surgeons for each video and to the individual scores of each surgeon. Ethical committee approval was obtained (2023.008.IRB2.006), and informed consent was given by each patient.

### 2.4. Statistical Analysis

Statistical analysis was performed using the Python programming platform (version 3.9). The scores from the surgeons were first tested for normality using the Shapiro–Wilk test with an alpha level of 0.05. As the scores were found not to be normally distributed, the intraclass correlation coefficient (ICC) method was chosen to assess the interobserver and intraobserver agreements [27].

To evaluate the interobserver agreement among the scores given by three surgeons, the ICC method based on the mean rating (k = 3), absolute agreement, and 2-way random effects model was leveraged. On the other hand, a 2-way mixed-effects model with absolute agreement was employed on the consecutive evaluations of the surgeons to compute the intraobserver agreement [28].

In addition to the assessment of intra- and interobserver agreements, the agreement between the system outputs and the average surgeon scores (both for all scores and score subgroups) was evaluated under the following scenario: the average surgeon scores were used as one score vector and the system outputs as the second. This comparison was again conducted using ICC, with a mean rating model (k = 2) with absolute agreement in a two-way random-effects framework.

## 3. Results

**Figure 3:**
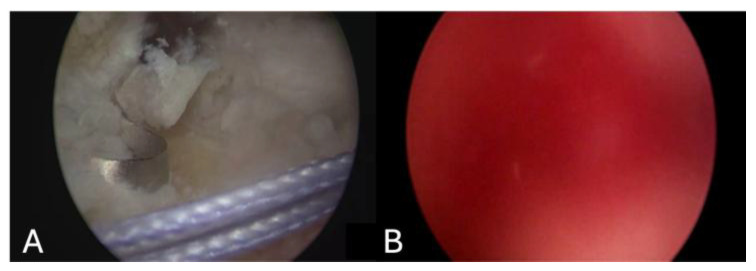
Comparison of scores 1 (**A**) and 10 (**B**) based on the scores of the fourth surgeon. These scores were verified by the system as the lowest (1) and the highest (9.46) scores of the whole series.

**Figure 4:**
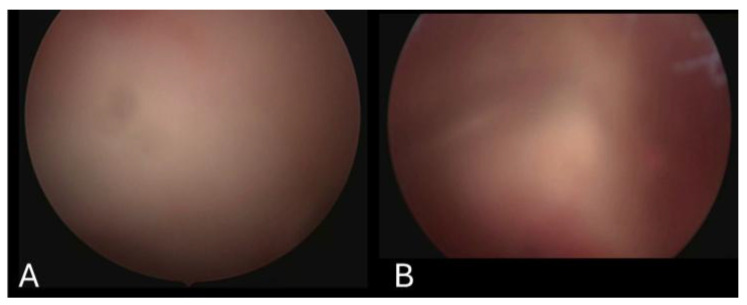
Arthroscopic images extracted from video clips scored as 3.17 (**A**) and 4.08 (**B**) by the system. In image (**A**), the background inflammation was not classified as bleeding, resulting in a low score of 3.

**Figure 5:**
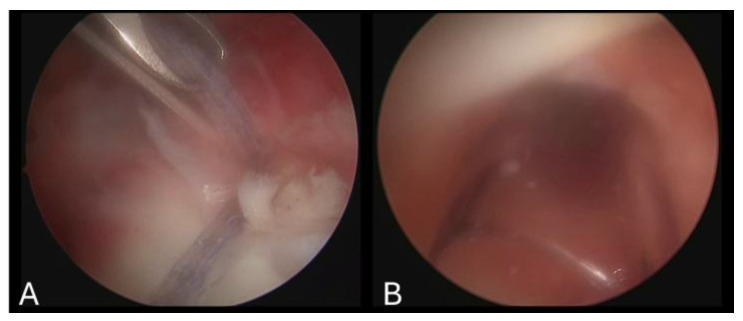
Arthroscopic images extracted from video clips scored as 4.21 (**A**) and 5.3 (**B**) by the system. Both images were uniformly scored as 4 by all three surgeons, demonstrating the system’s ability to distinguish between similar images.

Arthroscopic rotator cuff repair video records belonging to 15 patients were retrospectively reviewed to create 200 video clips. The mean and standard deviation of the three surgeons’ scores were calculated to be (5.55 ± 2.80), (4.87 ± 2.89) and (4.88 ± 2.34). Then, the average score of the three surgeons for each video was calculated and noted as the ‘Surgeon Score’. The average value of the ‘Surgeon Score’ was 5.10 and ranged between 1 and 9.66. The system scored the videos from 1 to 9.46 and the average score was 5.08. When each score from 1 to 10 was considered as a subgroup, the number of videos that were distributed into the subgroups by both the surgeons and the system was comparable. Sample images from the different subgroups are presented in Figure 3, Figure 4 and Figure 5.

**Figure 6:**
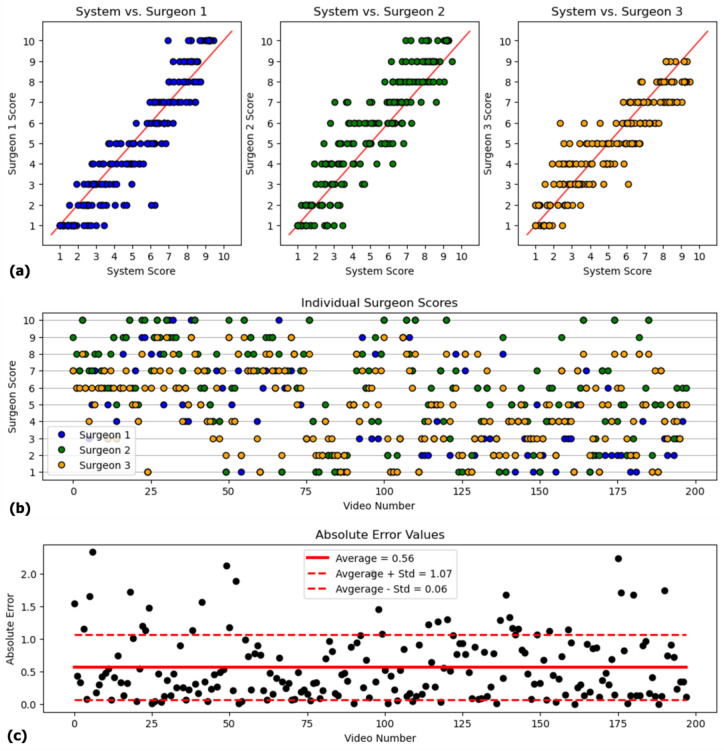
(**a**) Individual surgeons’ scores against the system-generated scores. (**b**) Individual scores of each surgeon for 200 video clips. (**c**) Absolute errors for 200 video clips.

Accordingly, the interobserver reliability of the three surgeons was found to be 0.96 with a 95% confidence interval of [0.93, 0.97], revealing that the interobserver reliability was in excellent agreement (ICC > 0.9). In a similar manner, intraobserver agreement for the consecutive evaluations was calculated to be 0.97, demonstrating excellent consistency among each rater’s evaluations. The scatter plots in Figure 6a illustrate the correspondence between the system’s scores and those assigned by each surgeon (Surgeons 1, 2, and 3). The clustering of data points along the diagonal line across all three graphs demonstrates a high degree of concordance between the system and the surgeons, further validating the system’s scoring reliability. However, the observed horizontal variance in score distributions also indicates inherent subjectivity in the surgeons’ scoring processes. This difference is also evident in Figure 6b, which displays the individual scores assigned by each of the three surgeons. The MAE values for each surgeon across all subgroups, in comparison to the system-derived scores, are provided in Supplementary Material Table S1.

The mean absolute error between the surgeon score and the system was calculated for each video and its average was found to be 0.56 and the standard deviation was 0.50, yielding >90% accuracy. When the agreement between the system outputs and the average surgeon scores was assessed, an agreement ranging from [0.96, 0.98] was obtained, with a confidence level of 96.7% (ICC = 0.967). The model’s performance for individual videos is illustrated in Figure 6c, where the MAE values are plotted for each sample. The majority of errors lie within the mean ± std range of [0.06, 1.07], with a few outliers exceeding 2.0, indicating potential challenges in handling certain edge cases that require further investigation. Despite these outliers, the model demonstrates consistent performance with no observable trends in degradation, underscoring its reliability and robustness for general use.

Additionally, the system’s performance was evaluated for each score subgroup to delineate the interval most accurately scored by the system (Table 1). Maximum absolute error was noted at the 3.5–5.5 interval, while the best congruence was demonstrated between 5.5 and 8.5. Accordingly, smaller ICC scores were observed within the 3.5–5.5 interval. It should be noted that the ICC scores for individual subgroups were lower compared to the overall ICC score, likely due to the reduced number of data points within each subgroup.

## 4. Discussion

### 4.1. Comparison with the Existing Literature

This study aimed to validate a video image processing system’s ability to recognize and accurately quantify bleeding in arthroscopic surgery. According to our results the system is proven to mimic the human eye perception of bleeding by a mean absolute error of 0.56. The mean values scored by the surgeons and the system were calculated to be 5.10 and 5.08, respectively, which demonstrated very high coherence.

The identification of bleeding using visual technology has been previously implemented in laparoscopic surgery [21,22,23]. Computer vision algorithms have predominantly been used in laparoscopic surgery, considering the fact that inadvertent bleeding can be life-threatening in the abdomen. Contrarily, arthroscopic surgery is considered a safe procedure in terms of blood loss, while bleeding can hinder surgeon’s performance by obscuring visual clarity and thus prolonging the procedure itself. The appearance of bleeding differs between laparoscopic and arthroscopic systems as well. In laparoscopy, blood presents as a more uniform texture in an inflated environment, making visual recognition simpler [24]. In contrast, blood in arthroscopy acts like a red dye, continuously dissolving into the arthroscopic fluid, which complicates image processing. Basically, all shades of the red color spectrum can be observed during an arthroscopic procedure depending on the intensity of bleeding and the rate of the clear inflow. At this point, it can be speculated that RGB (**r**ed, **g**reen, **b**lue) alone would not be sufficient to discern various shades within the red spectrum, and more than one color metric would be necessary to accurately identify intra-articular bleeding during arthroscopy.

Implementing more than one color metric is the essential value we added to Tuijthof et al.’s work, who were the first to utilize the method of the estimation of bleeding through the proportion of red pixels in the arthroscopy images in 2011 [25]. They evaluated the effectiveness of three types of pump systems in establishing a clear view in shoulder arthroscopy using only the RGB color space and defined the ‘bleeding episode’ as when the proportion of red pixels exceeds 20%. Unfortunately, the RGB color space alone cannot capture the full range of red shades, making it ineffective in accurately detecting the rate of bleeding into the joint. Additionally, Tuijthof et al.’s system primarily focused on detecting acute, significant bleeding events or “bleeding episodes”, likely overlooking lower-intensity, continuous oozing into the joint. A red blurry vision due to a multifocal, slow but steady bleeding is more troublesome and difficult to control than a single acute discharge. This is why we implemented a gradient-based scoring system and aimed to distinguish small changes at the low levels, where we believe the interventions against bleeding mostly affect.

Barnes et al. very recently proposed a resembling computational metric assessment that aimed to automatically quantify the image quality [26]. Similar to ours, their primary goal was to establish an objective scoring system to improve visualization; however, the two methods differ in many technical aspects. Barnes et al. analyzed the general image quality metrics including brightness, local contrast, and image entropy along with the redness for representing the blood. Our system can be considered rather focused on bleeding for accurately grading the arthroscopic image from 1 to 10 concerning solely the blood in the joint. To enhance the accuracy of blood detection, we employed two color spaces known to better mimic human perception—HSV and CIELAB—during thresholding and integrated saturation values to further consolidate the system’s performance [19,20]. Contrarily, Barnes et al.’s method utilized only the RGB space and lacked thresholding, which is crucial in discerning red artifacts such as inflammation. Moreover, we selected 3 s videos to provide homogeneity within the video clip to increase the accuracy in its evaluation. The mean video length of 11 s used in Barnes’ study could manifest an important visual variation compromising the coherence between surgeon rating and computational image metrics. Finally, we believe that the effect of the measures taken against bleeding could not be detectable on a four-point scale from poor to excellent view. In our study, we requested the surgeons to rate the bleeding from 1 to 10 and, as expected, obtained significantly high coherence among their scores. Hence, our results support Barnes’ work in terms of high reliability among fellowship trained shoulder surgeons in assessing arthroscopy images. 

**Figure 7:**
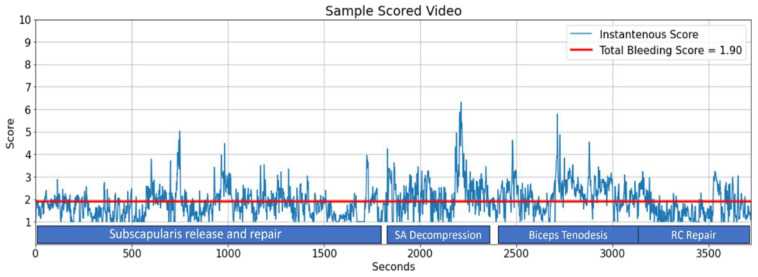
The system’s analysis report of a 62 min procedure. The total bleeding score is a novel parameter that indicates the average bleeding score of this arthroscopic rotator cuff repair procedure and calculated as 1.90/second. Ersin et al. previously reported that the occurrence of bleeding varies along different stages of an arthroscopic shoulder procedure [11]. Indeed, the total bleeding score is highest in subacromial decompression (2.28) and biceps tenodesis (2.26) phases of the surgery, while it is lowest in the final phase where the rotator cuff tendons are repaired (1.59).

Since the interventions to reduce bleeding are continuous and affect the whole procedure, the efficacy of these external factors must be evaluated throughout the procedure. To underrate a generally well-visualized procedure, bearing in mind only a remarkably hemorrhaging subacromial decompression, is a very common error of subjective scoring systems. The method described in this study introduces a ‘total bleeding score’ that is defined as the sum of each second’s score of the video divided by its total length (Figure 7). Thus, a single numerical score could be attributed to an entire surgical procedure as well as to its specifically targeted phases for an objective comparison to others. Another advantage of this score is that it is independent of the length of the procedure and helps the surgeons in the standardization of their data.

Finally, we evaluated the performance of our model by comparing it across two distinct scenarios, which are summarized as follows:

1. We included a comparison with a baseline computer vision (CV) algorithm and incorporated the use of the RGB color space. While the overall pipeline described in Algorithm 1 remained unchanged, lines 5 and 6 were modified to extract the R, G, and B components (specifically focusing on the R channel) instead of the H, S, V and L, A, B color parameters. The modified algorithm was executed under the same conditions, resulting in an absolute error of 1.43 ± 0.84, with an ICC of 0.86 (which is lower than our system’s ICC of 0.97).
2. We prepared a batch of images by taking a clean arthroscopy frame and augmenting it with a red overlay, where the transparency varied dynamically between 10% and 90%. The prepared images were then used as test inputs for the proposed algorithm. Some of these sample images are provided as Supplementary Material Figure S2. With 90% transparency corresponding to a score of 1 and 10% transparency corresponding to a score of 9, the system achieved an absolute error of 2.26 ± 1.25 and an ICC of 0.82. The relatively lower performance was anticipated, as the artificially generated images did not accurately represent the complex tissue and bleeding dynamics (such as saturation, lightness, hue, etc.) but instead reflected solely the change in redness. Since our algorithm is specifically optimized to detect dynamic changes in addition to redness, these results highlight that the algorithm does not rely exclusively on redness but also incorporates other key parameters (such as saturation, lightness, hue, etc.), reinforcing its robustness and adaptability to more realistic scenarios.

Based on these results, our model demonstrated superior performance compared to the baseline tasks, with lower mean absolute errors and fewer outliers, highlighting its ability to generalize effectively across diverse scenarios.

### 4.2. Limitations and Future Work

Both our study and system have limitations of their own that need to be emphasized. Despite their substantial experience in arthroscopic surgery, comparing the system’s results to only three evaluators may sound insufficient. However, we managed this weakness by increasing the number of video clips and by repeating the evaluation with a two-week interval. High inter- and intraobserver reliability suggested that the results of the three fellowship trained surgeons may be generalized.

One of the limitations of the study lies in the arthroscopic treatment of humeral tuberosity fractures. The fracture surfaces often share the same color as blood, which can confuse the system. However, since fractures suitable for arthroscopic interventions are rare, this limitation can be considered negligible. For the current system, we recommend excluding these interventions until artificial intelligence is integrated to accurately recognize fractures. Additionally, this work primarily focused on rotator cuff repair procedures, which involve the extensive debridement of well-vascularized soft tissue and result in significantly more bleeding compared to other procedures. Hence, future work will focus on incorporating artificial intelligence to expand the system’s capability to handle diverse procedures beyond rotator cuff repairs, as well as addressing edge cases and external noise, thereby enhancing generalizability.

In terms of computational efficiency, the current system takes 0.416 s to process and score each of the 3 s video clips. This shows that our current system would also be applicable to real-time scoring scenarios. One significant direction involves incorporating this objective framework into arthropump systems used in arthroscopic procedures. This integration will aim to enhance the effectiveness of these systems by minimizing fluid displacement into surrounding soft tissues and reducing operative time while ensuring consistently clear visualization throughout the procedure. We believe that such a system can potentially alleviate the surgeon from the task of pressure adjustment by automatically fine-tuning the pressure level based on the bleeding score obtained from the proposed image processing system.

### 4.3. Clinical Implications

The ultimate goal of this innovative study was to provide a reliable measurement tool for comparing different interventions that aim to reduce intra-articular bleeding and maintain a clear vision during arthroscopic procedures. Nevertheless, the ability of accurately detecting bleeding and objectively quantifying the degradation of the arthroscopic image can have exciting expansions. In a digitalized world where many systems operate independent of human supervision, it will be possible to train a computerized pump system to simultaneously evaluate the video during the operation and intervene autonomously in case of bleeding to increase the inflow. With the help of a gradient-based scoring system with high precision, an automatic pump can react faster than the surgeon and can titrate the inflow just enough to improve the image (reduce the score), thus potentially avoiding extravasation due to unnecessarily high pressures. The realization of this technology clearly requires a numerical interplay between the arthroscopic image and the computerized system, which is provided by the method described in our study.

We acknowledge that future research is necessary to test our system under different circumstances and adjust it accordingly. Nevertheless, considering more than 90% accuracy in imitating human perception, we occasionally had difficulties deciding whether the system is being tested or if it is the golden standard itself.

## 5. Conclusions

Despite significant advancements in arthroscopic visualization technology, such as 4K resolution and advanced image enhancement, even minimal bleeding can disrupt procedures and diminish the benefits of these innovations in reducing operative time. Current research on bleeding control methods remains limited by the absence of objective measures to assess clinically meaningful improvements.

In this context, the system presented in our study has been demonstrated to closely mimic human eye perception of bleeding, achieving a mean absolute error of 0.56 and a high coherence, with a mean score of 5.08 compared to the surgeons’ score of 5.10. This method enables surgeons to accurately quantify and compare the effectiveness of interventions aimed at bleeding control. Furthermore, our system allows for the objective establishment of the relationship between intraoperative arterial blood pressure and visual clarity, offering new insights into optimizing surgical conditions and the chemical composition of arthroscopic irrigation fluids. Additionally, the integration of a gradient-based numerical scoring system into autonomous arthropump devices will enable the real-time adjustment of inflow rates during procedures, enhancing surgical precision and efficiency while minimizing extravasation caused by unnecessarily high pressures.

## 6. Patents

The authors wish to disclose that they have applied for a patent for the technology described in this manuscript. The patent application is currently under review, and the process is ongoing.

## Figures and Tables

**Table 1:** Mean absolute errors and standard deviations of the system evaluated for each score subgroup. Note that the system is stronger between 5.5 and 8.5 and weaker between 3.5 and 5.5.

Rater Score Average	System Score	Absolute Error	ICC Score
s < 1.5	1.64 ± 0.65	0.54 ± 0.57	0.74
1.5 ≤ s < 2.5	2.23 ± 0.59	0.48 ± 0.38	0.83
2.5 ≤ s < 3.5	3.03 ± 0.67	0.53 ± 0.39	0.81
3.5 ≤ s < 4.5	3.84 ± 1.13	0.87 ± 0.58	0.63
4.5 ≤ s < 5.5	4.75 ± 0.91	0.73 ± 0.51	0.72
5.5 ≤ s < 6.5	6.21 ± 0.69	0.45 ± 0.49	0.89
6.5 ≤ s < 7.5	6.88 ± 0.52	0.34 ± 0.26	0.90
7.5 ≤ s < 8.5	7.98 ± 0.64	0.43 ± 0.41	0.89
8.5 ≤ s < 9.5	8.63 ± 0.69	0.64 ± 0.54	0.76
9.5 ≤ s	9.08 ± 0.24	0.59 ± 0.24	0.68

## Data Availability

The data presented in this study, including the system’s scores, surgeons’ evaluations, and arthroscopy video clips, are available upon request from the corresponding author. The code developed by the researchers is currently unavailable due to ongoing patenting procedures but may be shared upon approval once the patenting process is complete.

## Supplementary Materials

The following supporting information can be downloaded at: https://www.mdpi.com/article/10.3390/jimaging11020040/s1. Supplementary Material Figure S1. Another set of examples from scores 1 to 9, based on the scoring of the fourth surgeon who selected the images from the arthroscopy records; Supplementary Material Table S1. Comparison of the mean absolute errors and standard deviations of the individual surgeon scores with respect to the system score; Supplementary Material Figure S2. A clean arthroscopy frame was taken and augmented with a red overlay, where the transparency varies dynamically between 10% and 90%. The artificial images were then used as test inputs for the proposed algorithm.
