# Supplementary material for: Validation of Novel Image Processing Method for Objective Quantification of Intra-Articular Bleeding During Arthroscopic Procedures
Source: J Imaging. 2025 Jan 31;11(2):40. doi: 10.3390/jimaging11020040 (PMC11856628; doi:10.3390/jimaging11020040)
Supplement: Supplementary file 1 [file jimaging-11-00040-s001.zip › Supplementary Material 1.pdf]

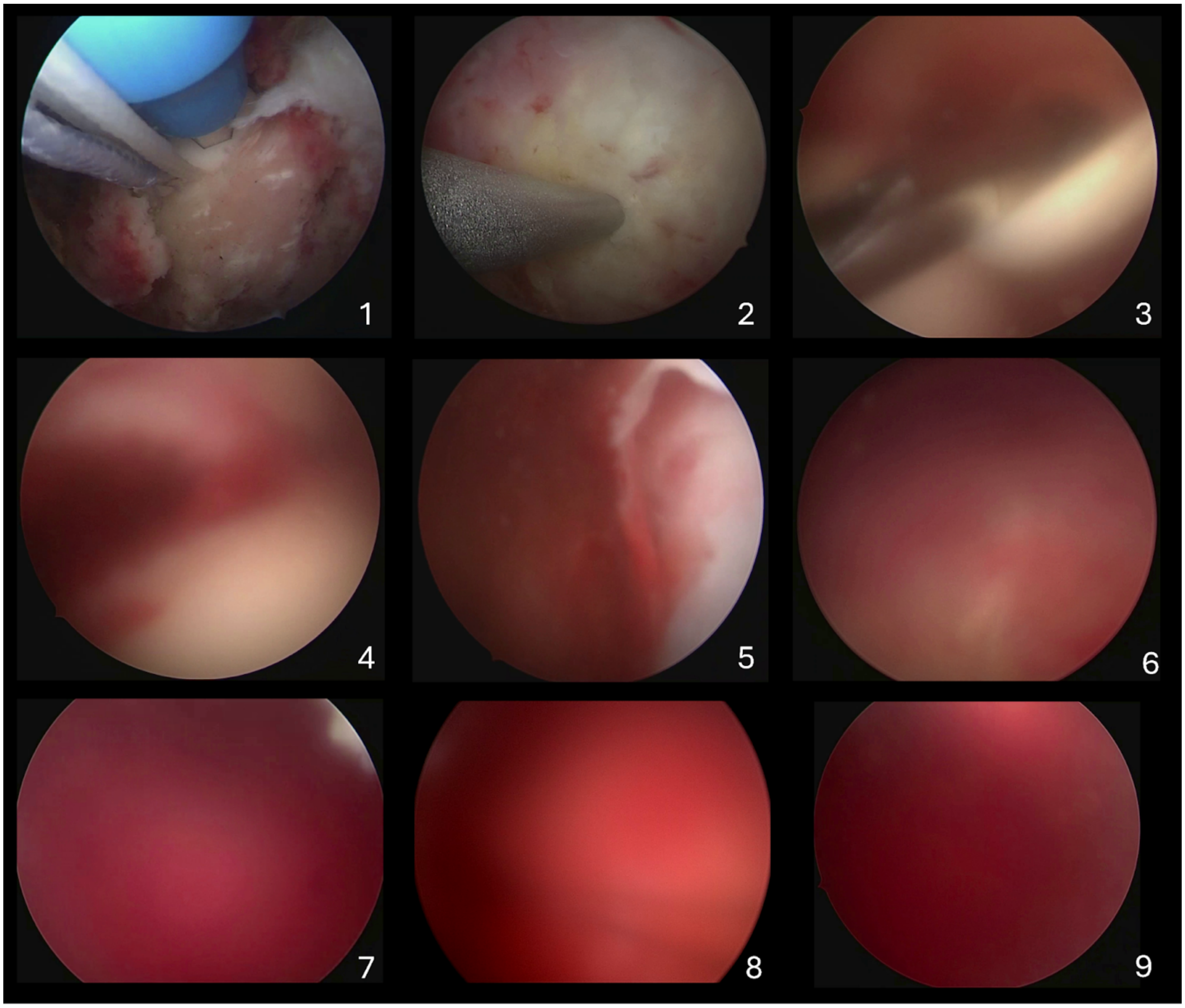

Supplementary Material 1. Another set of examples from scores 1 to 9, based on the scoring of the fourth surgeon who selected the images from the arthroscopy records.
