# Supplementary material for: Validation of Novel Image Processing Method for Objective Quantification of Intra-Articular Bleeding During Arthroscopic Procedures
Source: J Imaging. 2025 Jan 31;11(2):40. doi: 10.3390/jimaging11020040 (PMC11856628; doi:10.3390/jimaging11020040)
Supplement: Supplementary file 1 [file jimaging-11-00040-s001.zip › Supplementary Material 2.pdf]

Supplementary Material 2. Comparison of the mean absolute errors and standard deviations of the individual surgeon scores with respect to the system score.

System Score	Absolute Error for Surgeon 1	Absolute Error for Surgeon 2	Absolute Error for Surgeon 3
**s < 1.5**	0.24 ± 0.18	0.31 ± 0.26	0.54 ± 0.32
**1.5 ≤ s < 2.5**	0.66 ± 0.34	0.73 ± 0.64	0.98 ± 0.80
**2.5 ≤ s < 3.5**	0.76 ± 0.62	1.29 ± 0.84	0.74 ± 0.59
**3.5 ≤ s < 4.5**	0.82 ± 0.50	1.37 ± 0.97	0.79 ± 0.52
**4.5 ≤ s < 5.5**	0.93 ± 0.74	0.79 ± 0.66	0.77 ± 0.47
**5.5 ≤ s < 6.5**	0.94 ± 0.98	0.94 ± 0.71	0.81 ± 0.66
**6.5 ≤ s < 7.5**	0.83 ± 0.66	1.02 ± 0.84	0.79 ± 0.49
**7.5 ≤ s < 8.5**	0.89 ± 0.55	0.90 ± 0.59	0.80 ± 0.53
**8.5 ≤ s < 9.5**	0.82 ± 0.21	0.82 ± 0.31	1.08 ± 0.56
**9.5 ≤ s**	*comparison unavailable since the maximum system score was 9.5		
