# Supplementary material for: Validation of Novel Image Processing Method for Objective Quantification of Intra-Articular Bleeding During Arthroscopic Procedures
Source: J Imaging. 2025 Jan 31;11(2):40. doi: 10.3390/jimaging11020040 (PMC11856628; doi:10.3390/jimaging11020040)
Supplement: Supplementary file 1 [file jimaging-11-00040-s001.zip › Supplementary Material 3.pdf]

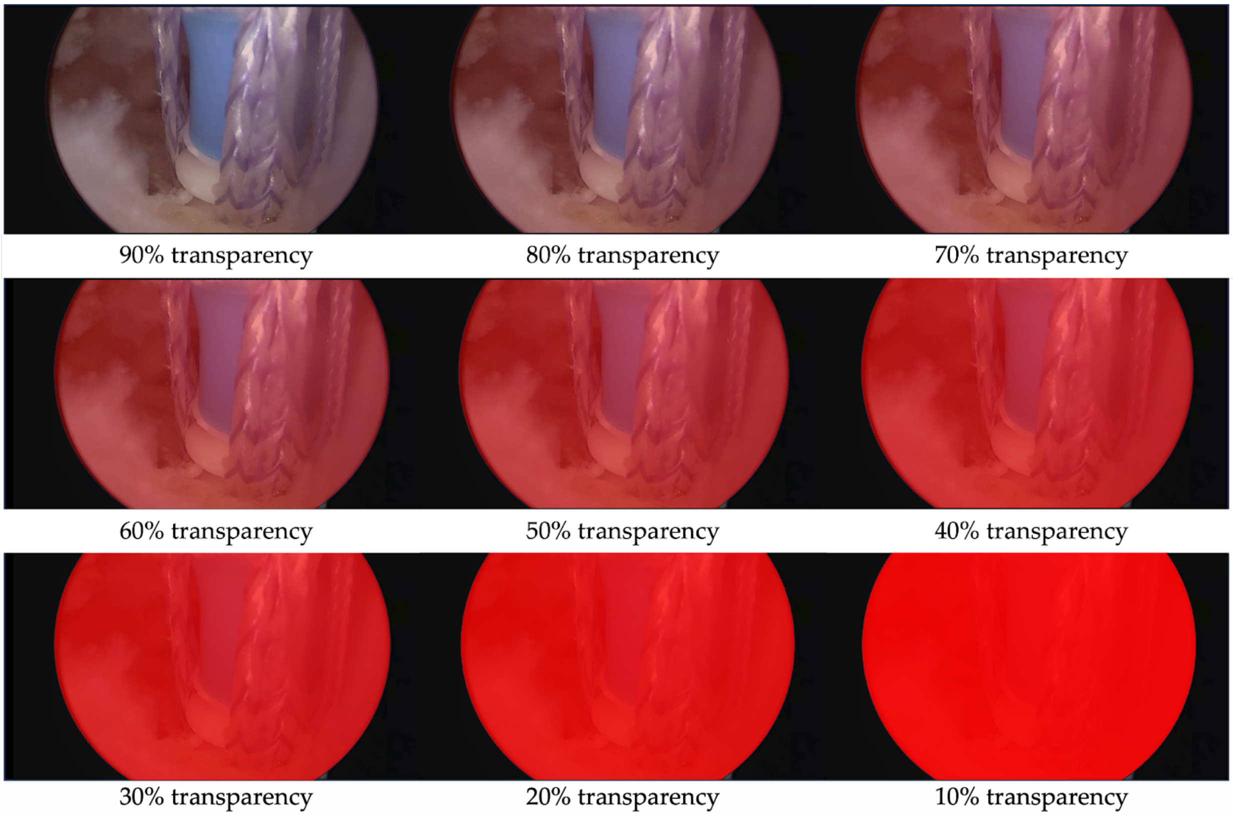

Supplementary Material 3. A clean arthroscopy frame was taken and augmented with a red overlay, where the transparency varies dynamically between 10% and 90%. The artificial images were then used as test inputs for the proposed algorithm.
